# Simultaneous monitoring of mouse grip strength, force profile, and cumulative force profile distinguishes muscle physiology following surgical, pharmacologic and diet interventions

**DOI:** 10.1038/s41598-022-20665-y

**Published:** 2022-09-30

**Authors:** Joseph J. Munier, Justin T. Pank, Amie Severino, Huan Wang, Peixiang Zhang, Laurent Vergnes, Karen Reue

**Affiliations:** 1 Department of Molecular, Cellular, and Integrative Physiology, University of California, Los Angeles, CA 90034 USA; 2 Department of Human Genetics, David Geffen School of Medicine at UCLA, 695 Charles E. Young Drive South, Los Angeles, CA 90095 USA; 3 Department of Psychiatry and Biobehavioral Disease, Semel Institute for Neuroscience and Human Behavior, David Geffen School of Medicine at UCLA, Los Angeles, CA 90095 USA; 4 Molecular Biology Institute, University of California, Los Angeles, CA 90095 USA

**Keywords:** Risk factors, Ageing, Metabolism, Neurophysiology, Biomarkers, Metabolic disorders, Neurological disorders, Musculoskeletal system, Behavioural methods, Biological techniques, Software

## Abstract

Grip strength is a valuable preclinical assay to study muscle physiology in disease and aging by directly determining changes in muscle force generation in active laboratory mice. Existing methods to statistically evaluate grip strength, however, have limitations in the power and scope of the physiological features that are assessed. We therefore designed a microcontroller whose serial measure of resistance-based force enables the simultaneous readout of (1) peak grip strength, (2) force profile (the non-linear progress of force exerted throughout a standard grip strength trial), and (3) cumulative force profile (the integral of force with respect to time of a single grip strength trial). We hypothesized that muscle pathologies of different etiologies have distinct effects on these parameters. To test this, we used our apparatus to assess the three muscle parameters in mice with impaired muscle function resulting from surgically induced peripheral pain, genetic peripheral neuropathy, adverse muscle effects induced by statin drug, and metabolic alterations induced by a high-fat diet. Both surgically induced peripheral nerve injury and statin-associated muscle damage diminished grip strength and force profile, without affecting cumulative force profile. Conversely, genetic peripheral neuropathy resulting from lipin 1 deficiency led to a marked reduction to all three parameters. A chronic high-fat diet led to reduced grip strength and force profile when normalized to body weight. In high-fat fed mice that were exerted aerobically and allowed to recover for 30 min, male mice exhibited impaired force profile parameters, which female mice were more resilient. Thus, simultaneous analysis of peak grip strength, force profile and cumulative force profile distinguishes the muscle impairments that result from distinct perturbations and may reflect distinct motor unit recruitment strategies.

## Introduction

A decline in grip strength in humans and mice has been associated with all-cause mortality and diseases such as type 2 diabetes, cardiovascular disease, respiratory disease, and cancer^1–3^. Increasing evidence supports the use of grip strength in general health assessments, particularly in aging adults, as a predictor for future bone and muscle function, as well as cognitive decline and dementia^1,4,5^. The establishment of reliable quantitative methods for grip strength assessment in preclinical models is a critical step towards understanding mechanisms underlying impaired muscle function in disease states and development of targeted pharmacotherapies to prevent this decline.

The laboratory mouse, *Mus musculus*, can support its body weight using only the fore paws or hind paws, such that grip force measurements on front paws provide a relevant metric for strength^6^. Traditional methods for grip strength measurement in the mouse employ spring scales to report peak grip strength^7^. This approach provides a readout of peak grip strength exerted at a single time point, which limits the utility of the assay and requires larger sample sizes during study design^8^. Instruments that allow digital readout of peak grip strength represent improvements on the spring scale but can be cost-prohibitive. And although some digital force-sensitive resistor-based sensors are capable of reporting high-fidelity, repeated-measures analyses of grip strength^9^, this type of analysis has not been routinely or rigorously employed. Alternative methods to assess muscle performance include the wire hang test, which evaluates force with respect to time^10^, or treadmill running, which measures endurance^11^. These techniques are typically low-to-moderate throughput and are difficult to translate to the grip force measurements routinely used in clinical settings.

We propose a method that allows simultaneous, reproducible, and high-throughput measurement of three parameters of muscle strength: peak grip strength, force profile (the time-course analysis of individual grip strength readings), and cumulative force profile (the integrated product of force over the time course of an individual reading). To perform these measurements, we designed our device to enable detection of applied force in a repeated measures design. This has several advantages over existing methods. The repeated measures capability improves statistical power, increases confidence in ascertaining the true peak applied force, and enables detection of alterations in the applied force during the short period of time that the measure is being recorded. The difference between these techniques is analogous to the difference between using a video vs. a photograph to analyze a golf swing. A video provides a deeper information set about the swing trajectory than are apparent from a single frame. Clinicians and scientists also appreciate the difference between a repeated-measures analysis of plasma glucose, as opposed to a single area under the curve measure of glucose clearance. Analogously, our device enables analysis of dynamic elements that influence grip strength that may inform about distinct properties of muscle physiology that are relevant to common causes of muscle impairment.

In healthy individuals, we expect that grip strength and force profile should be highly correlated. Indeed, peak grip strength and cumulative force profile are often used interchangeably to report about muscle^12,13^ or neural physiology^14–16^. However, it is appreciated that muscle impairment likely differs depending upon the disease context. For example, aging is known to cause a shift towards recruitment of larger motor units, potentially to compensate for decreased firing rate, and thus lower generated force^17,18^. Conversely, in post-acute neurological deficits such as stroke, there is failure of motor unit recruitment^19^. We hypothesize that simultaneous measurement of peak grip strength, force profile, and cumulative force profile will provide insight into distinct aspects of muscle function and impairment in disease.

Here, we describe an open-source instrument that allows the simultaneous measurement of peak grip strength, force profile and cumulative force profile. We assessed these parameters in mouse models with different types of muscle and nerve pathology induced surgically, genetically, pharmacologically, or by metabolic dysregulation. We detected distinct relationships between grip strength, force profile, and cumulative force profile in the individual pathological states that we assessed. Our findings illustrate unique signatures in muscle (dys)function that occur in disease states, which likely reflect the distinct molecular mechanisms that underlie these pathologies.

## Methods

### Animals

Animal studies were performed in accordance with the Guide for the Care and Use of Laboratory Animals and after approval by the UCLA Institutional Animal Care and Use Committee. All experiments were performed in accordance to ARRIVE guidelines. Animals were housed in a 12 h light:dark cycle at ambient temperature with ad libitum access to food, water and environmental enrichment (nesting material). Mice were handled for 5 min on 3 consecutive days prior to study onset to acclimate them to moderate handling and to minimize stress.

Sciatic nerve entrapment study: 11–13 week-old male and female C57BL/6J mice (The Jackson Laboratory, Bar Harbor, ME; stock 0065, n = 6–8/group/sex) were group-housed (3/cage). Sciatic nerve entrapment surgeries to produce mechanical allodynia were performed similarly to those previously described^20^, with some modifications. Briefly, animals were deeply anesthetized with isoflurane and the left hind leg was isolated, shaved, and prepared for sterile surgery. Forceps were used to separate, but not stretch or tear, the rectus femoris and biceps femoris posterior muscles. The muscles were separated and opened to a 3 mm length with care to not stretch or damage the sciatic nerve. A presterilized 2 mm polyethylene cuff was cut, gently guided, and placed onto the sciatic nerve. The cuff was secured around the nerve but able to slide up and down without pinching. The muscle tissue was then closed, concealing the nerve and cuff. Animals were given 0.5 g of acetaminophen in peanut butter per cage for 2 days post-operatively. Grip strength was performed 3–5 weeks following recovery from surgery.

Genetic peripheral neuropathy study: *Lpin1*^–/–^ mice on BALB/cByJ genetic background were derived from breeders originally obtained from The Jackson Laboratory (strain #001592) and group housed (2–4/cage). *Lpin1*^–/–^ mice were compared to *Lpin1*^+/+^ littermates (n = 4/group).

Statin diet study: 16-week old female C57BL/6J mice (n = 5) were group housed (2–3/cage) and fed a diet containing simvastatin (0.1 g/kg simvastatin, Research Diets D11060903i, New Brunswick, NJ) or corresponding control diet without simvastatin.

High-fat diet study: 12 week-old male and female C57BL/6J mice were group housed (n = 6/group/sex; 2/cage) and fed a diet with 60% of kcal from fat (S3282, Bio-Serv, Flemington, NJ) for 11 weeks.

### Mechanical paw withdrawal threshold and Von Frey filament tests

Mechanical paw withdrawal thresholds were determined via two methods. For sciatic nerve entrapment studies, 50% paw withdrawal thresholds was determined with Von Frey filaments as previously described^21,22^. For studies examining the longitudinal effect of statin diet on grip strength, an automated dynamic plantar aesthesiometer (Ugo Basile, IT) was used. Animals were given at least 30 min to acclimate to the testing environment before recording to minimize variability in recordings.

### Grip strength measurements

#### Grip strength apparatus

**Figure 1:**
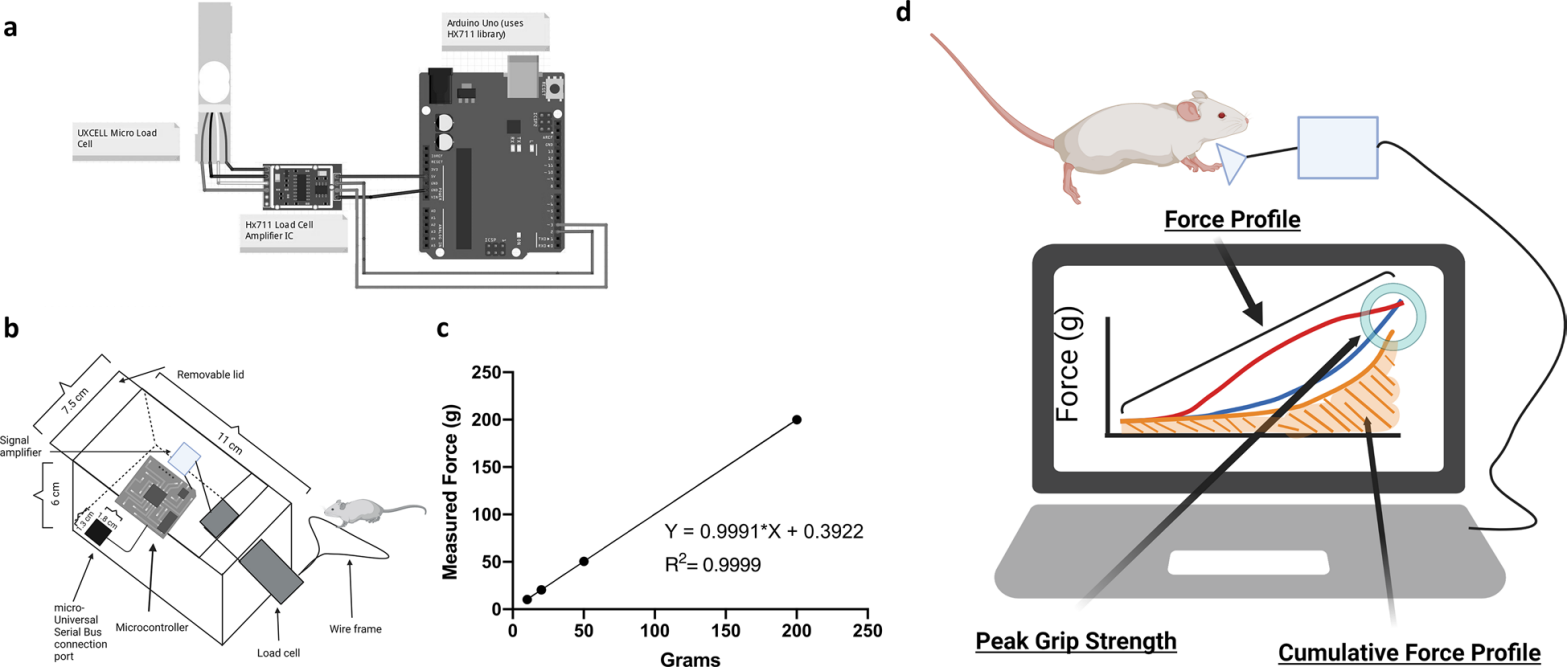
Microcontroller enables simultaneous measure of three distinct grip parameters. (**a**) Schematic representing the configuration of a force-sensitive resistor cell (UXCELL Micro Load Cell) to an amplifier (Hx711 Load Cell) that uses a microcontroller (Arduino) to interface with any OSX/Windows operating system. (**b**) View of the external apparatus housing containing the circuit. Power is connected with micro-universal serial bus port. Removable allows access to adjust circuit as needed. (**c**) Linear regression of the detected force in response to check weights. Applied force is highly linear within the typical dynamic range of grip strength measurements (****p < 0.0001, Pearson’s Correlation, R^2^ = 0.9999). (**d**) Illustration displaying the detection of peak grip strength (highest point for force), force profile (the shape of the line), and cumulative force profile (the area under the force profile curve). The three lines represent hypothetical curves obtained for three distinct mouse subjects. The blue line illustrates the force profile of a control or wild-type mouse without physiological perturbation, and the highest point shows the maximum grip strength. The yellow line represents the force profile of a mouse with reductions in both force profile and peak grip strength. The red line illustrates the force profile of a mouse with a similar peak grip strength as the control animal, but with a distinct force profile that suggests abnormal compensation to achieve the same peak grip strength.

The grip strength apparatus consisted of an Hx711 load cell (Bolsen Tech), amplifier (SparkFun Electronics), and microcontroller (Arduino) housed in a 3D-printed polyethylene container (available in Suppl. File 1). Calibration and weight detection scripts are provided in Suppl. File 2. A schematic of the apparatus is provided in Fig. 1. The apparatus was programmed to record the force applied to the load cell approximately every 1 decisecond and calibrated to a 100 g check-weight prior to each recording session. Animals were given a set of 5 grip strength trials each trial day. Since variability in performance can occur throughout the trial (e.g. if the mouse fails to grasp firmly with both forepaws or other technical errors,) a normative claim (good vs. bad) was used for each recording. Good readings (average = 3) for each trial day were aligned and averaged for each mouse.

We noted several important considerations for attaining consistent measurements. The individual conducting the assay should remain calm throughout the procedure to limit social transfer of stress to subjects. It is advisable to practice the procedure on trial subjects prior to making initial measurements on experimental cohorts, and for the same individual to perform all measurements within an experimental cohort to avoid variations related to measurement technique or to characteristics of the handler that are detected by mice (odor, demeanor)^23^. To perform a measurement, the mouse is grasped firmly, but gently, at the base of the tail and allowed to grip the bar handle of the apparatus (which mice do instinctively). The mouse is positioned perpendicular to the bar handle as force is applied by gently pulling the mouse in a fluid motion at a speed of ~ 3 cm/s. The mouse will grasp for ~ 1 s as force is applied. Analysis of recorded values can be segmented to give 3 separate measures:

1. Peak grip strength: the highest recorded value normalized to value _peak–10_.
2. Force profile: normalized and aligned two or three-way repeated measures ANOVA analysis of grip force applied.
3. Cumulative force profile (∫Force(t)dt): the integrated product of force with respect to time during a single trial.

### Body composition and glucose tolerance

Body composition was measured in conscious mice using an EchoMRI instrument (EchoMRI LLC, Houston, TX). Glucose tolerance tests were performed as we have previously^24^. Briefly, 20% d-glucose dissolved in sterile 0.9% NaCl was delivered i.p. at 10 µL/g body weight in mice that had been fasted 5 h (0800–1300). Glucose levels were measured via AlphaTrak glucometer immediately prior to glucose injection and at hourly intervals until plasma glucose returned to baseline.

### Treadmill measurements

Mice were familiarized with and trained to the treadmill apparatus by training sessions on three separate days. Testing was performed at a speed of 8 m/min with an increase of 1 m/min every minute^11^. Trials were terminated when a mouse spent ≥ 5 s in the “danger zone” (the origin at the base of the treadmill lane). After 30 min of recovery, peak grip strength, force profile and cumulative force profile were measured to assess how recovery from aerobic exertion affected these parameters.

### Statistical analyses

Data are expressed as mean ± SEM. Interactions between variables (sex, diet, time, treatment) were performed using 3-way repeated measures ANOVA. Post-hoc tests were computed with Tukey’s multiple comparison test or Sidak’s multiple comparison for repeated measures two-way ANOVA. All statistical analyses were performed using Prism 9 (GraphPad) with an alpha cutoff of 0.05.

## Results

### Microcontroller enables simultaneous measurement of three distinct grip parameters

After first-hand experience in the technical issues that can arise when employing grip strength as a preclinical assay, we sought to design a low-cost instrument that allows simultaneous assessment of multiple muscle strength parameters. The instrument (Fig. 1a–c) couples a force-sensitive resistor, digital amplifier, and a digital microcontroller to enable high-fidelity, linear, repeated-measures data of single grip strength bouts. The measured force of the apparatus is linear with respect to known check weights (Fig. 1c). As illustrated in Fig. 1d, the output of the measurements includes the peak grip strength (highest value on the y-axis), the force profile (the time-dependent application of force during a grip strength trial), and the cumulative force profile (the integrated product of the force profile curve).

### Differential effects of localized and generalized peripheral nerve impairment on muscle strength parameters

**Figure 2:**
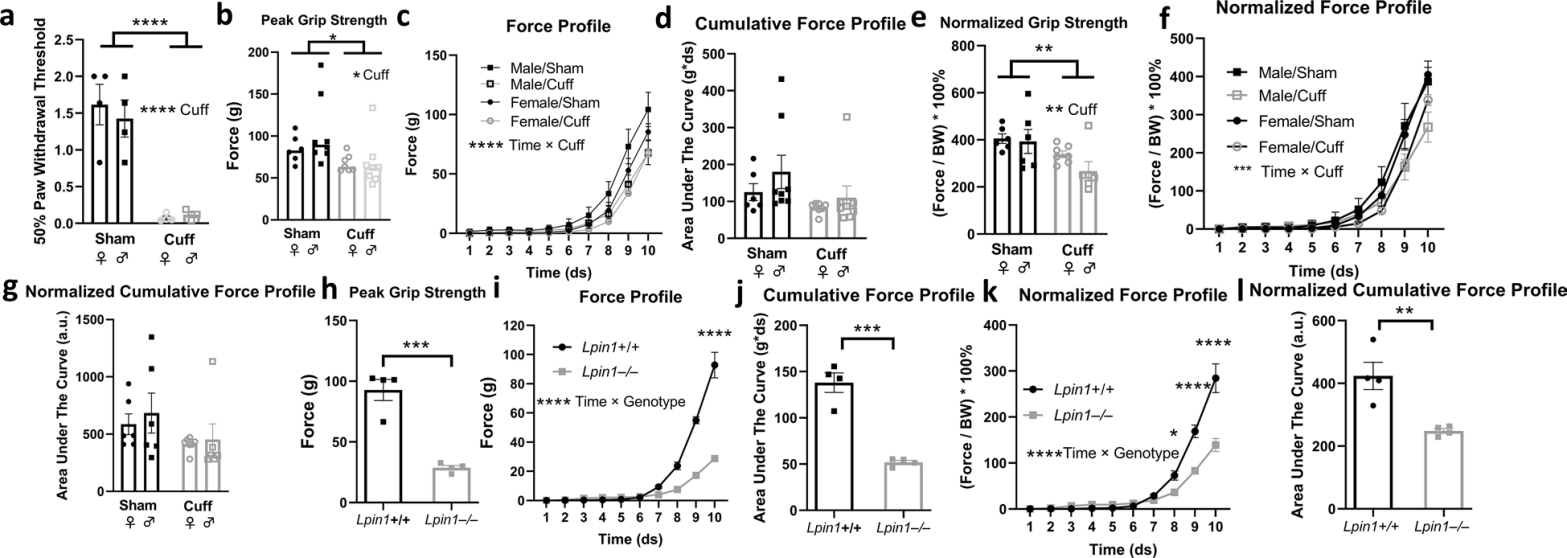
Differential effects of localized (**a**–**g**) and generalized peripheral nerve impairment (**h**–**l**) on muscle strength parameters. (**a**) Following recovery from sciatic nerve entrapment surgery (cuff), paw withdrawal thresholds are reduced (****p < 0.0001, Student’s t-test). (**b**) Peak grip strength is reduced in cuffed animals (*p < 0.05, Two-way ANOVA). (**c**) Force profile is reduced in animals exposed to cuff in both sexes. (****p < 0.0001, time × cuff interaction, Tukey’s multiple comparison three-way ANOVA; Female/Sham n = 6, Male/Sham n = 8, Female/Cuff n = 7, Male/Cuff n = 8). (**d**) Cumulative force profile is unchanged in either sex following cuff. (**e**) Normalizing grip strength to the body weight (BW) does not alter the differences seen following cuff. (**f**) Force profile normalized to BW is reduced in cuffed animals (****p < 0.0001, time × cuff interaction, Tukey’s multiple comparison three-way ANOVA Female/Sham n = 6, Male/Sham n = 6, Female/Cuff n = 7, Male/Cuff n = 6). (**g**) BW-normalized cumulative force profile is unchanged following cuff. (**h**,**i**) *Lpin1*^–/–^ animals have reduced grip strength, force profile(**p < 0.01, Student’s t-test; *p < 0.05, **p < 0.01, ****p < 0.0001, time × genotype interaction, two-way repeated measures ANOVA; n = 4/group) and (**j**) cumulative force profile (***p < 0.001, Student’s t-test). (**k**,**l**) *Lpin1*^–/–^ mice exhibit reduced force profile and cumulative force profile after BW normalization (****p < 0.0001, time × genotype interaction two-way repeated-measures ANOVA; **p < 0.01, Student’s t-test).

Peripheral nerve injury is expected to diminish grip strength, and it has been demonstrated previously that female mice exhibit reduced grip strength in response to sciatic nerve entrapment^25^. We sought to evaluate the effect of peripheral nerve injury by sciatic nerve entrapment through measurements of grip strength, force profile, and cumulative force profile in male and female mice. We verified the efficacy of nerve entrapment by determining that compared to sham controls, both male and female mice exhibited the expected reductions in peripheral sensitivity as demonstrated by paw withdrawal threshold in the von Frey filament test (Fig. 2a). Sciatic nerve entrapment also led to reduced grip strength and force profile in both sexes (Fig. 2b,c), but did not significantly alter cumulative force profile (Fig. 2d). This remained true after normalization to body weight (Fig. 2e–g).

We also evaluated a genetic model of generalized peripheral neuropathy, the *Lpin1*^–/–^ (lipin 1–deficient) mouse, which exhibits impaired peripheral nerve function due to alterations in Schwann cell lipid synthesis^26^. Similar to the nerve entrapment model of localized peripheral nerve damage, *Lpin1*^–/–^ mice exhibited marked reductions in grip strength and force profile. Only generalized peripheral neuropathy, however, led to reduced cumulative force profile (Fig. 2h–j). These alterations were also evident after normalization to body weight (Fig. 2k,l).

### Acute statin treatment impairs grip strength and force profile, but not cumulative force profile

**Figure 3:**
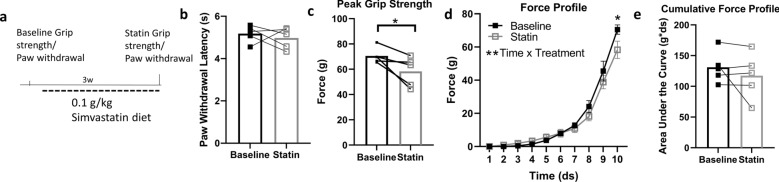
Acute statin treatment impairs grip strength and force profile, but not cumulative force profile. (**a**) Experimental design for assessing statin-associated muscle symptoms in female C57BL/6 J mice. (**b**) Statin treatment does not affect mechanical sensitivity, as determined by automated paw withdrawal thresholds. (**c**,**d**) Statin-treated mice have reduced grip strength and force profile (*p < 0.05, paired t-test, Tukey’s multiple comparison; **p < 0.01 time × treatment interaction, two-way repeated measures ANOVA; n = 5/group). (**e**) Statin treatment does not alter cumulative force profile.

Statin drugs are known to induce muscle pain and weakness in some individuals, and these adverse effects are more widely reported in women than in men^27–31^. Except in cases of severe muscle damage, statin-induced muscle weakness is typically reported in a subjective manner, without quantitative measurements. We assessed grip strength, force profile, cumulative force profile, as well as paw withdrawal threshold, in female mice at baseline prior to statin treatment, and after feeding a diet containing 0.1% simvastatin for 3 weeks (Fig. 3a). Statin did not alter paw withdrawal threshold (Fig. 3b), indicating that short-term statin treatment did not induce peripheral mechanical hyperalgesia. Statin diet administration for 3 weeks led to reduced grip strength and force profile (Fig. 3c,d), without altering cumulative force profile. (Fig. 3e). This demonstrates the sensitivity of the apparatus to detect subtle changes in muscle function in a longitudinal study, and to detect changes in specific muscle parameters (e.g., grip strength, force profile) independent of others (e.g., cumulative force profile).

### High-fat diet imposes sex-specific effects on metabolism and muscle performance

Diet-induced obesity is a common cause of impaired glucose homeostasis and risk for type 2 diabetes^32,33^. Obesity is also associated with an accumulation of intramyocellular lipids, which may impact glucose uptake by muscle and contribute to impaired skeletal muscle function^34–38^. High-fat meals can also induce acute inflammatory responses^39^. We sought to determine whether diet-induced obesity is associated with changes in muscle strength and recovery after exercise, and whether sex influences these parameters.

**Figure 4:**
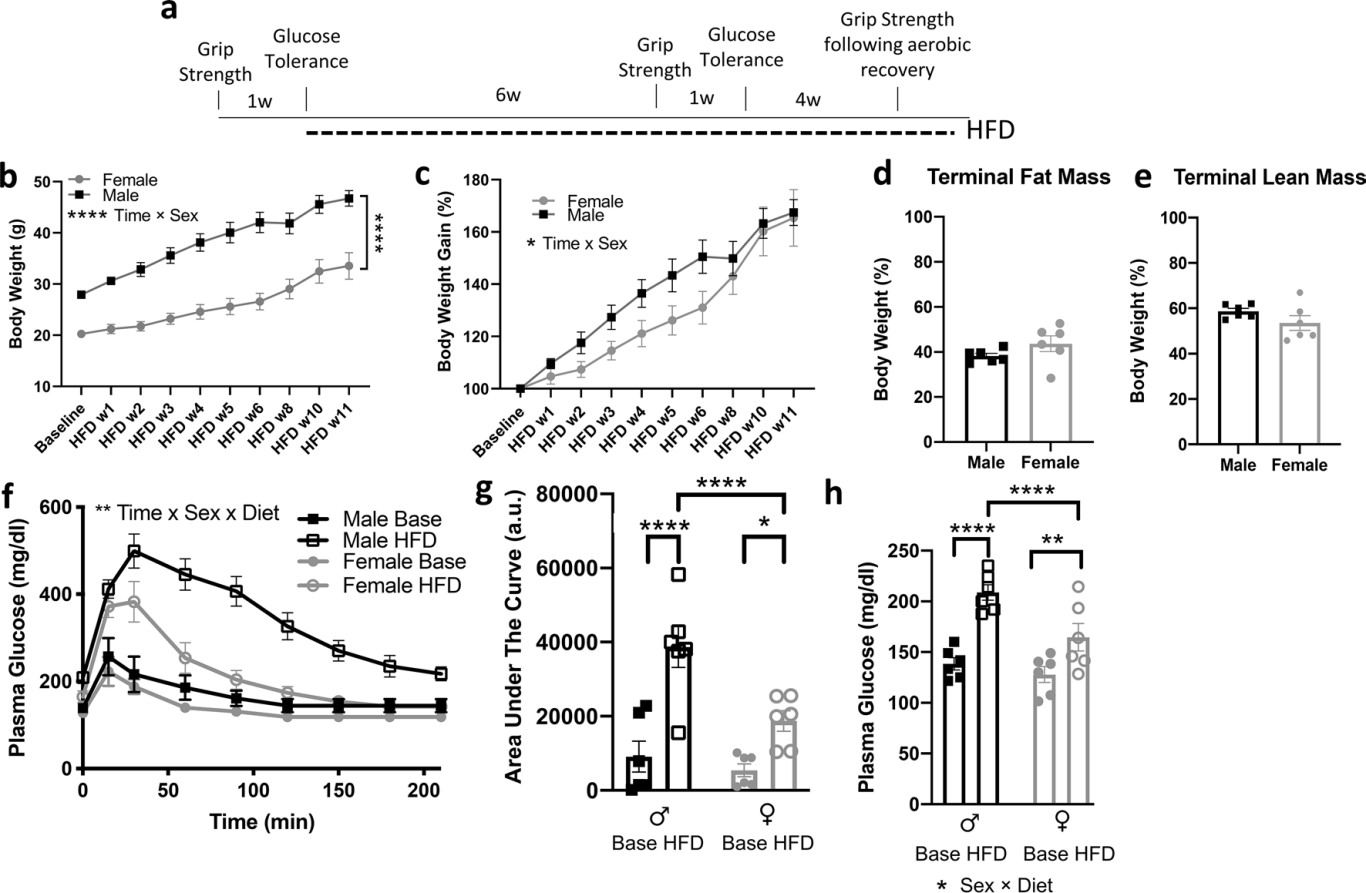
Diet-induced weight gain and glucose intolerance in males and females. (**a**) Experimental design for high-fat diet (HFD) administration and assessment of metabolic and muscle parameters. (**b**) Raw body weight of animals across experimental timeline (****p < 0.0001 time × sex interaction, two-way repeated measures ANOVA; n = 6/sex). (**c**) Body weight gain in male and female mice fed HFD (*p < 0.05, time × sex interaction, two-way ANOVA). (**d**,**e**) Similar proportional fat and lean mass in HFD males and females. (**f**) Glucose tolerance curves in males and females at baseline (Base) and following HFD. Males show greater glucose intolerance than females in response to HFD (**p < 0.01, time × sex × diet interaction, three-way repeated measures ANOVA). (**g**) Integrated glucose clearance is significantly increased following HFD, and higher in males compared to females (*p < 0.05, ****p < 0.0001, two-way ANOVA, Tukey’s multiple comparison post-hoc test). (**h**) Plasma glucose is elevated in response to HFD compared to baseline (measured following a 5 h fast), and higher in males than females (**p < 0.01, ****p < 0.0001; *p < 0.05, sex × diet interaction, Tukey’s post-hoc test, two-way ANOVA).

We characterized metabolic parameters (body weight, body composition, fasting glucose levels and glucose tolerance) and grip strength measures in male and female C57BL/6J mice fed a chow diet and in the same mice after 11 weeks of HFD (Fig. 4a). Males had larger body weight throughout the entire experiment (Fig. 4b). Males and females experienced similar increases in proportional body weight in response to HFD after 11 weeks, but the kinetics were slightly different between sexes. Males rapidly gained weight and plateaued, whereas females lagged behind in the first weeks, but ultimately reached a similar proportional weight gain as males (Fig. 4c). After 11 weeks of HFD, the proportional fat and lean mass was similar for males and females (Fig. 4d,e).

Consistent with expected effects of chronic HFD feeding, males and females exhibited impaired glucose tolerance compared to their baseline levels on chow diet (Fig. 4f,g). In addition, both male and female mice exhibited elevations in fasting glucose levels on HFD compared to their baseline values (Fig. 4h). Males had higher absolute levels of glucose intolerance and fasting glucose than female mice (Fig. 4f–h), consistent with previous reports in young male vs. female C57BL/6J mice fed HFD^40^.

**Figure 5:**
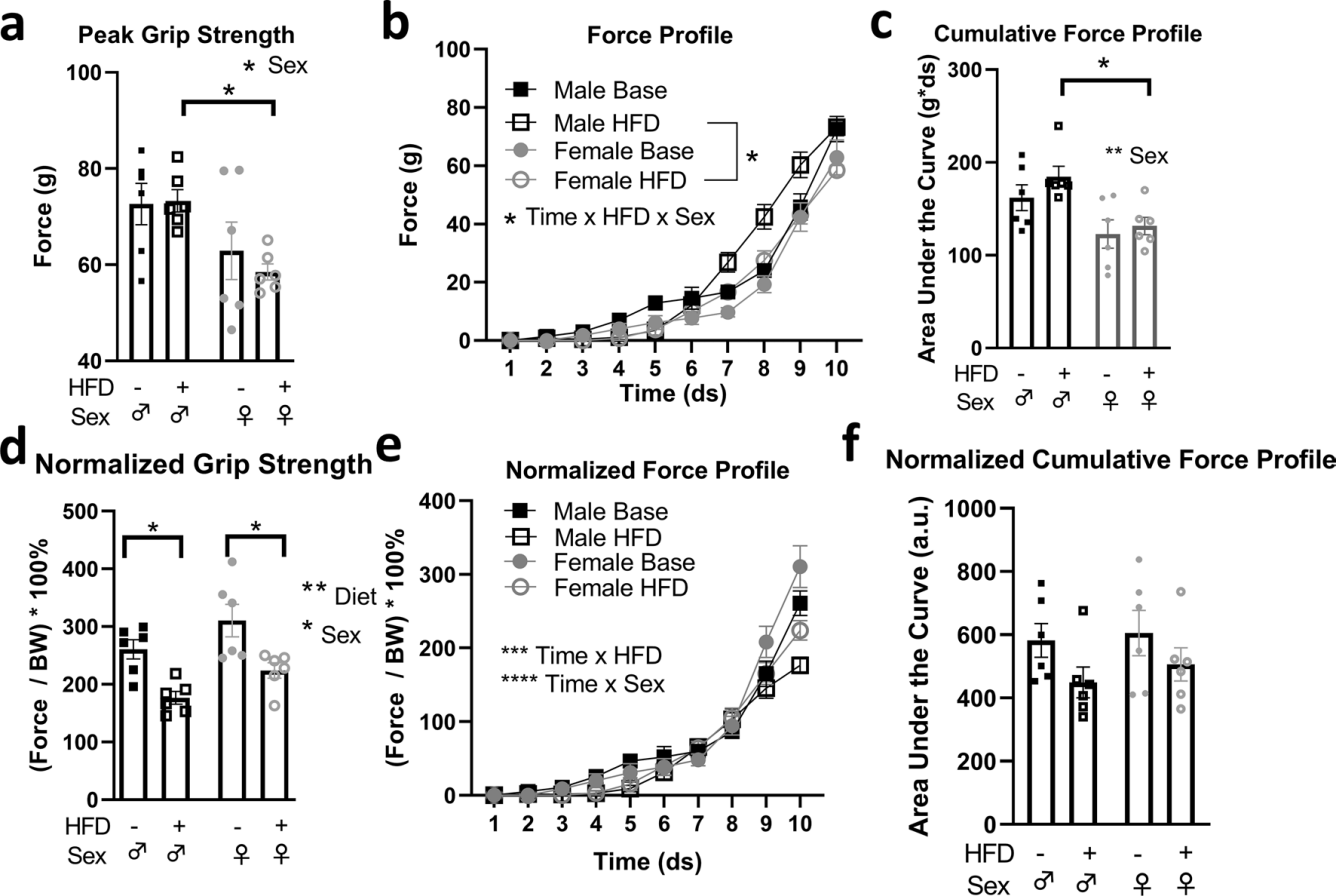
Sex differences in diet-induced alterations in muscle strength. (**a**,**b**) Females have reduced peak grip strength following high fat diet (HFD) exposure. Males have a HFD-induced increase in force profile (*p < 0.05, time × HFD × sex interaction, Tukey’s post-hoc multiple comparison, three-way, repeated measures ANOVA; n = 6/group). (**c**) Males exposed to HFD have significantly greater cumulative force profile compared to females. (*p < 0.05, **p < 0.01, effect of sex, two-way repeated-measures ANOVA, Sidak post-hoc test). (**d**) Peak grip strength normalized to body weight is reduced in both sexes following HFD exposure (* p < 0.05, Two-way, repeated measures ANOVA). (**e**) Normalized force profile, but not (**f**) normalized cumulative force profile, is reduced by HFD (***p < 0.001, time × HFD interaction; ****p < 0.0001, time × sex interaction; three-way, repeated-measures ANOVA).

We measured grip strength parameters at baseline and after 6 weeks of HFD. At 6 weeks of HFD, we identified an effect of sex on grip strength and cumulative force profile, with females showing lower values for both parameters compared to their male counterparts (Fig. 5a,c). However, these differences were at least partially accounted for by body weight, as normalization to body weight revealed similar reductions to grip strength in females and males, and similar cumulative force profiles between the sexes (Fig. 5d,f). Force profile was altered by HFD in a sex-dependent manner (Fig. 5b; time × HFD × sex interaction, p < 0.05), but this was negated after normalization to body weight (Fig. 5e).

### Recovery of grip strength following aerobic training in HFD-fed mice is sex-dependent

**Figure 6:**
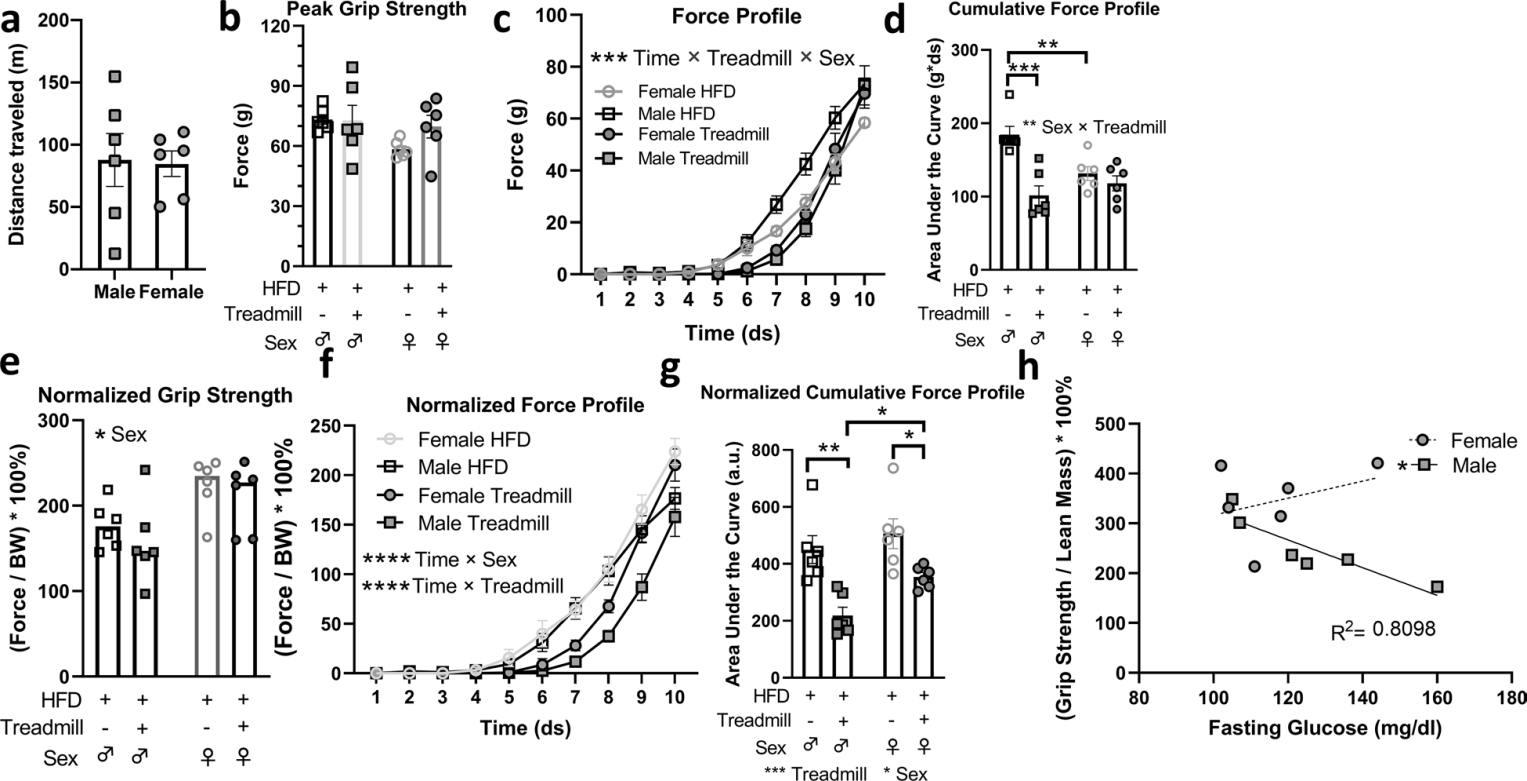
Acute recovery from aerobic exercise reduces force profile in HFD male mice. (**a**,**b**) Distance travelled during a treadmill fatigue test, and grip strength following 30 min recovery from treadmill exertion, are similar between sexes. (**c**) Force profile is selectively reduced in males following recovery from treadmill exposure (***p < 0.001, time × treadmill × sex interaction, Tukey’s post-hoc multiple comparison, three-way, repeated measures ANOVA; n = 6/group). (**d**) Cumulative force profile is selectively reduced in males during acute recovery (**p < 0.01, sex × treadmill interaction, ***p < 0.001, two-way repeated measures ANOVA, Sidak post-hoc test). (**e**) Body-weight normalized peak grip strength is not influenced by treadmill recovery. (**f**) Force profile normalized to body weight are altered by both sex and treadmill exposure (****p < 0.0001, time × sex and time × treadmill, Tukey’s post-hoc multiple comparison, three-way, repeated measures ANOVA). (**g**) Females are moderately resilient to treadmill exposure mediated-reductions in cumulative force profile normalized to body weight (*p < 0.05, ***p < 0.01, two-way repeated measures ANOVA, Sidak post-hoc test). (**h**) Fasting glucose levels and grip strength normalized to lean mass are negatively correlated following treadmill fatigue in male mice, but not female mice (*p < 0.05, coefficient of correlation = 0.8098).

It has been shown that both stress and fatigue may influence grip strength^41–43^. We assessed grip strength parameters in mice made obese by HFD that underwent treadmill exertion, recovered for 30 min, and were measured for strength parameters. The HFD-fed male and female mice traveled a similar distance on the treadmill prior to exertion (Fig. 6a). After the treadmill recovery period, both sexes displayed grip strength similar that before the treadmill, indicating a sufficient recovery period after exercise (Fig. 6b). However, there was a sex difference in force profiles following aerobic recovery, with males showing a lag in force profile during recovery (Fig. 6c). Additionally, there was a significant sex × exercise interaction on cumulative force profile, with only males exhibiting a reduction in cumulative force profile during recovery (Fig. 6d). When values were normalized to body weight, females showed higher peak grip strength values than males both before and after exercise (Fig. 6e). Normalized force profile was altered by both sex and treadmill exposure, with females showing higher peak values and being less influenced by recovery from aerobic exercise (Fig. 6f). The normalized cumulative force profile was reduced in both sexes (Fig. 6g), but females recovered to a greater degree than males (Fig. 6f,g). Finally, we assessed the potential relationship between grip strength and glucose levels following exercise and recovery. We identified a male-specific correlation between maximal grip strength (normalized to lean mass) and fasting plasma glucose levels following the fatigue test (R^2^ = 0.8098; Fig. 6h).

## Discussion

Muscle grip strength measurements have been used in humans and in preclinical models to predict all-cause mortality and multiple pathologies^1–5^. However, measurements in preclinical models are subject to limitations in reproducibility and in the parameters assessed. Here, we utilized our novel apparatus to measure maximal grip strength, force profile, and cumulative force profile simultaneously in several mouse models of impaired muscle and/or nerve function. The repeated measures design of our data output improves statistical power, providing the possibility to reduce the number of animals needed for complex study designs^44^. The open-source code and detailed design scheme of our instrument make this device accessible at a low cost. Most importantly, the simultaneous measurement of grip strength, force profile and cumulative force profile will enable more detailed analysis of muscle function than is possible with traditional methods to evaluate grip strength.

Our analysis of grip strength, force profile, and cumulative force profile revealed distinct patterns for mouse models with muscle impairment of distinct etiologies (summarized in Table 1). We found that reduced grip strength and force profile in mice with surgically induced peripheral nerve damage was sex-independent and occurred without alterations in cumulative force profile. On the other hand, a genetic model of generalized peripheral neuropathy showed reductions in all three parameters**.** We also detected decrements in muscle function during short-term (3 week) treatment with statin drug. Specifically, statin induced reductions in grip strength and force profile, while maintaining cumulative force profile. Given that human statin drug courses typically last for months or years, further studies may examine whether longer courses of statin treatment promote further reductions in grip strength and/or alterations in force profile. These findings also raise the possibility that assessing human distal grip strength could provide an objective clinical measure of statin adverse effects on muscle.

**Table 1:** Summary of alterations in grip strength, force profile, and cumulative force profile following surgical, pharmaceutical and dietary interventions.

Condition		Grip strength	Force profile	Cumulative force profile	Normalized grip strength	Normalized force profile	Normalized cumulative force profile
Peripheral nerve injury		↓	↓	⇿	↓	↓	⇿
Peripheral neuropathy		↓	↓	↓	↓	↓	↓
Statin-associated myopathy		↓	↓	⇿	N/A	N/A	N/A
High-fat diet	Treatment	⇿	♂↑	⇿	↓	↓	⇿
	Sex	♀ < ♂	♀ < ♂	♀ < ♂	♀** > **♂	♀** > **♂	⇿
Aerobic recovery	Treatment	⇿	♂↓	♂↓	⇿	↓	↓
	Sex	⇿	♀** > **♂	♀** > **♂	♀** > **♂	♀** > **♂	♀** > **♂

↓, decrease, ↑, increase, or ⇿, unchanged following intervention relative to control. ♂ > ♀, male values greater than female values; difference following intervention. ♀ > ♂, female values greater than male values; difference following intervention. ♂↓ Male-specific reduction. ♂ ↑ Male-specific increase.

Finally, we assessed the effects of metabolic stress (HFD-induced obesity) and exercise stress on muscle strength parameters. Exposure to HFD is known to impair metabolic^45^ and muscle function^46^ in mice and humans, and acute exercise can decrease grip strength in mice^47^. High-fat diet did not alter peak grip strength in males or females, but did alter force profile exclusively in males. Furthermore, when strength parameters were normalized to body weight, the fat fed female mice showed greater grip strength and force profile than males. During recovery from aerobic exercise in chronically fat-fed mice, females appeared to be more resilient than males. Whereas males reduced their cumulative force profile during recovery, females maintained levels similar to those prior to exercise. Finally, fat-fed males showed a negative correlation between grip strength and fasting glucose levels, but females did not; it will be interesting to determine if this sex difference is associated with the sensitized recovery of skeletal muscle glycogen^48^ in females.

The identification of distinct muscle strength profiles for the various physiological states assessed here illustrate the utility of our instrument and the simultaneous analysis of grip strength, force profile, and cumulative force profile. Our experience also suggests that potential iterative improvements are possible in the standardization of temporally dynamic measures^49^, architectural design^50^, software, and hardware^9^. We also recognize selective areas that our device fails to improve on previous generations of grip strength methodology. Our use of a 1 s window recorded at 10 Hz was pragmatic based on the average duration of a single trial conducted by a single, trained experimenter. Others have pointed out ergonomic improvements to the collection of grip strength^50^ that could be employed with repeated-measures analysis. Use of multiple force-sensitive resistors (such as in the grip handle^9^) with improved dynamic ranges, accelerometers, and other features^51,52^ should theoretically improve functionality and/or reduce multiple forms of error. Novel, automated alignment tools^49^ may also be used to distinguish force profile signatures indicative of pathological states or user-error. Nevertheless, this instrument has provided replicable, nuanced measures of muscle strength that may be of valuable for numerous preclinical models.

In conclusion, the assessment of grip strength, force profile, and cumulative force profile simultaneously discriminates between muscle pathologies brought about by different causes (Table 1). Furthermore, direct comparisons of males and females revealed sex differences that merit consideration in the interpretation of previously published studies that assessed a single sex. Our findings lay the groundwork for future investigations into the unique molecular mechanisms underlying pathological changes to grip strength, force profile, and cumulative force profile. For example, it will be instructive to determine whether alterations in force profile and cumulative force profile result from altered motor unit recruitment through use of our apparatus coupled with in vivo electromyography. Additional studies may address the central and peripheral mechanisms that contribute to alterations in grip strength and/or force profile through simultaneous in vivo calcium imaging. Adaptation of methodology that assesses grip strength, force profile, and cumulative force profile simultaneously may enable a more holistic approach to characterizing neuromuscular pathology.

## Supplementary Information

Supplementary Information 1.

Supplementary Information 2.

## Data Availability

The datasets generated during and/or analyzed during the conduction of the present study are available from JJM and KR on reasonable request.
