# Supplementary material for: Simultaneous monitoring of mouse grip strength, force profile, and cumulative force profile distinguishes muscle physiology following surgical, pharmacologic and diet interventions
Source: Sci Rep. 2022 Sep 30;12:16428. doi: 10.1038/s41598-022-20665-y (PMC9525296; doi:10.1038/s41598-022-20665-y)
Supplement: Supplementary file 1 — Supplementary Information 1. [file 41598_2022_20665_MOESM1_ESM.pdf]

```
#include <HX711.h>    //Include the open-source library header, which has pre built functions to use the integrated circuit

#include "HX711.h"

#define DOUT  3     //define constants to represent the pins on the arduino (DOUT represents GPIO pin 3 on the arduino and CLK represents pin 2)
#define CLK  2

HX711 scale(DOUT, CLK);
// create an HX711 scale object using the 2 pins as input variables. See the library documentation for details

float calibration_factor = -600.0;
// define a calibration factor variable. Use this with a known calibration weight to properly calibrate the scale. Calibration factor ensures accurate weight readings from the load sensor.


void setup() {
  Serial.begin(9600);
// begin communication over serial interface at a baud rate of 9600 bits per second

  Serial.println("HX711 calibration sketch");
  Serial.println("Remove all weight from scale");
  Serial.println("After readings begin, place known weight on scale");
  Serial.println("Press + or a to increase calibration factor");
  Serial.println("Press - or z to decrease calibration factor");

// display the strings on the serial monitor for human readability and interaction with the scale. Used to manually determine appropriate calibration factor using pre-measured check weights.

  scale.set_scale(); //function to set the scale (see HX711 documentation for further details)
  scale.tare(); //Reset the scale to 0

  long zero_factor = scale.read_average(); //define a variable to store the average scale zero factor
  Serial.print("Zero factor: ");
  Serial.println(zero_factor);
}

void loop() {

  scale.set_scale(calibration_factor);
// set the calibrated factor
```

```
  Serial.print("Reading: ");
  Serial.print(scale.get_units(), 1);
//read the measurement in grams
  Serial.print(" grams");
  Serial.print(" calibration_factor: ");
  Serial.print(calibration_factor);
//display the calibration factor
  Serial.println();

  if(Serial.available())
  {
    char temp = Serial.read();
    if(temp == '+' || temp == 'a')
      calibration_factor += 10;
    else if(temp == '-' || temp == 'z')
      calibration_factor -= 10;
  }
//if the user inputs a keystroke that is + or a OR - or z, increase or decrease the calibration factor accordingly, until the displayed weight matches the calibration weight. For example, if using a 1 gram calibration weight, adjust the calibration factor u til the monitor displays "1 grams"
}
```
