# Supplementary material for: Simultaneous monitoring of mouse grip strength, force profile, and cumulative force profile distinguishes muscle physiology following surgical, pharmacologic and diet interventions
Source: Sci Rep. 2022 Sep 30;12:16428. doi: 10.1038/s41598-022-20665-y (PMC9525296; doi:10.1038/s41598-022-20665-y)
Supplement: Supplementary file 2 — Supplementary Information 2. [file 41598_2022_20665_MOESM2_ESM.pdf]

```
#include "HX711.h"

#define calibration_factor -1070.0 //This value is obtained using the
SparkFun_HX711_Calibration sketch. Determine experimentally before each recording session.

#define DOUT  3
#define CLK  2
HX711 scale(DOUT, CLK);

void setup() {
  Serial.begin(9600);
  Serial.println("HX711 scale demo");

  scale.set_scale(calibration_factor);
// set the scale with the determined calibration factor
  scale.tare(); //Assuming there is no weight on the scale at start up, reset the scale to 0

  Serial.println("Readings:");
}

void loop() {
  Serial.print("Reading: ");
  Serial.print(scale.get_units(), 2); //scale.get_units() returns a float
  Serial.print(" grams");
  Serial.println();
//this loop continuously outputs the reading of the load sensor in grams, to the serial monitor
}
```
